# Generation of Luciferase-Expressing *Leishmania infantum chagasi* and Assessment of Miltefosine Efficacy in Infected Hamsters through Bioimaging

**DOI:** 10.1371/journal.pntd.0003556

**Published:** 2015-02-13

**Authors:** Juliana Q. Reimão, Jordana C. Oliveira, Cristiana T. Trinconi, Paulo C. Cotrim, Adriano C. Coelho, Silvia R. B. Uliana

**Affiliations:** 1 Departamento de Parasitologia, Instituto de Ciências Biomédicas, Universidade de São Paulo, São Paulo, Brazil; 2 Departamento de Moléstias Infecciosas e Parasitárias, Instituto de Medicina Tropical, Universidade de São Paulo, São Paulo, Brazil; Institut de Recherche pour le Développement, FRANCE

## Abstract

**Background:**

The only oral drug available for the treatment of leishmaniasis is miltefosine, described and approved for visceral leishmaniasis in India. Miltefosine is under evaluation for the treatment of cutaneous leishmaniasis in the Americas although its efficacy for the treatment of human visceral leishmaniasis caused by *Leishmania infantum chagasi* has not been described. Drug efficacy for visceral leishmaniasis is ideally tested in hamsters, an experimental model that mimics human disease. Luciferase has been validated as a quantitative tool for the determination of parasite burden in experimental leishmaniasis. However, there are no reports of luciferase detection in the model of progressive visceral leishmaniasis in hamsters. Therefore, the aims of this study were to generate recombinant *Leishmania infantum chagasi* expressing the luciferase gene (Lc-LUC), characterize the biological properties of this transgenic line as compared with the wild-type parasites and evaluate miltefosine effectiveness in Lc-LUC infected hamsters.

**Methodology/Principal Findings:**

A transgenic line containing a luciferase encoding gene integrated into the ribosomal DNA locus was obtained and shown to produce bioluminescence which correlated with the number of parasites. Lc-LUC growth curves and susceptibility to pentavalent antimony and miltefosine in vitro were indistinguishable from the wild-type parasites. The effectiveness of pentavalent antimony was evaluated in Lc-LUC infected hamsters through bioimaging and determination of Leishman Donovan Units. Both methods showed concordant results. Miltefosine was effective in the treatment of Lc-LUC-infected hamsters, as demonstrated by the reduction in parasite burden in a dose-dependent manner and by prolongation of animal survival.

**Conclusions/Significance:**

Luciferase expressing parasites are a reliable alternative for parasite burden quantification in hamsters with advantages such as the possibility of estimating parasite load before drug treatment and therefore allowing distribution of animals in groups with equivalent mean parasite burden. Miltefosine was effective in vivo in an *L. infantum chagasi* experimental model of infection.

## Author Summary

Studies to determine drug efficacy in experimental models of leishmaniasis involve several difficulties. Parasite quantification in tissues is generally done by techniques that are laborious and time consuming, such as limiting dilution and microscopic counting of amastigotes in imprinting of infected organs. To overcome some of these problems, we described in this study an experimental model of visceral leishmaniasis (VL) using parasites expressing luciferase. *Leishmania infantum chagasi*, the etiologic agent of VL in Latin America, was genetically manipulated to express the *luciferase* gene in order to produce light upon addition of luciferin. We inoculated these parasites in hamsters and, about one month post-infection, we detected light in the abdominal and/or pelvic region, indicating widespread infection. We compared the data obtained by bioluminescence with microscopic counting of amastigotes in imprinting of infected spleens and both methods showed concordant results. Aiming to validate our model to be used in drug effectiveness experiments, we performed the experimental treatment with pentavalent antimony, the first choice drug for VL treatment in Latin America. We also treated infected hamsters with miltefosine, the only oral drug used for leishmaniasis treatment and for which effectiveness against *L*. *infantum chagasi* infected hamsters had not yet been reported.

## Introduction

Visceral leishmaniasis (VL) is a neglected vector borne disease that manifests with fever, fatigue, weight loss, anemia and hepatosplenomegaly in humans. Untreated, VL is almost 100% fatal [1]. VL is transmitted by phlebotomine sand flies and is caused by *Leishmania infantum* and *Leishmania donovani*. *L*. *infantum chagasi* [2] is the etiological agent of VL in Latin America. *L*. *infantum* is found in the Mediterranean Basin, while *L*. *donovani* is prevalent in the Indian subcontinent, South Asia and East Africa [1].

Leishmaniasis chemotherapy is currently a major issue in disease management and there is pressing need for new drugs and/or new treatment regimes. Pentavalent antimony (Sb^V^) is the first line drug for VL treatment in Brazil and in many countries, in spite of its high toxicity. Due to parasite resistance, the use of antimonials was interrupted in some regions of India and replaced by miltefosine, the first and only oral agent available for leishmaniasis treatment [3]. Phase 4 studies demonstrated high success rates in the treatment of VL with miltefosine in that country [4].

Nonetheless, susceptibility to miltefosine is variable amongst *Leishmania* species and its efficacy in New World *Leishmania* infections is still a matter of investigation [5–8]. Miltefosine was employed successfully in *L*. *infantum*-infected hamsters in an early curative model [9] but, to the best of our knowledge, it has not been evaluated in a chronic VL model of *L*. *infantum chagasi* infection.

BALB/c mice and hamsters are the most commonly used VL animal models for drug and vaccine testing. *L*. *donovani* infection in mice results in early parasite replication followed by immunological control and subclinical infection, but it does not reflect the progressive disease observed in human VL. Infections of the Syrian golden hamster (*Mesocricetus auratus*), on the other hand, lead to hepato and splenomegaly, relentless increase in visceral parasite burden, progressive cachexia and, ultimately, death. These clinical and pathological findings are similar to the picture found in human and canine VL. However, research performed on hamsters is still limited due to the lack of reagents such as antibodies against cell markers and cytokines [10, 11].

Quantification of parasite burden in hamsters experimentally infected with VL causative species is generally obtained by classical methods, such as limiting dilution and/or microscopic counting of amastigotes in imprinting of infected organs, through the determination of Leishman Donovan units (LDU). These methods are laborious and time consuming. Attempts to overcome these difficulties include the determination of parasite burden based on reverse transcription and real-time PCR, for example [12], but this alternative is not devoid of difficulties. Therefore, the development of a technique allowing easy quantification of parasites in various tissues would be very useful.

Luciferase has been validated as a quantitative tool for the determination of parasite burden in experimental cutaneous leishmaniasis in vivo and *ex vivo* in tissue samples from *Leishmania amazonensis*-infected mice [13]. Luciferase transfected parasites were also used to quantify *L*. *infantum* in BALB/c infected mice [14]. However, there are no reports on quantitative determination of parasite burden through luciferase-detection in the model of progressive VL in hamsters.

Therefore, in this work we aimed at developing a transgenic line of *L*. *infantum chagasi* expressing luciferase, testing its application as a tool to evaluate drug efficacy in VL and evaluating miltefosine’s efficacy in *L*. *infantum chagasi* infections.

## Methods

### Ethics statement

Animal experiments were approved by the Ethics Committee for Animal Experimentation (Protocol CPE-IMT 2012/145) of the Instituto de Medicina Tropical of the University of São Paulo. The research adhered to the Brazilian Guidelines for Care and Utilization of Animals from the Conselho Nacional de Controle e Experimentacão Animal (CONCEA).

### Parasites

Wild-type *Leishmania (Leishmania) infantum chagasi* (Lc-WT) (MHOM/BR/1972/LD) promastigotes were grown in 25 cm^2^ tissue culture flasks containing M199 medium (Sigma-Aldrich, St. Louis, MO, USA) supplemented with 10% heat-inactivated fetal calf serum (FCS; Gibco Invitrogen Corporation, NY, USA), 0.25% hemin (Sigma-Aldrich) and 2% sterile male human urine at 25°C. Parasites were maintained in male golden hamsters (*Mesocricetus auratus*) and infections were performed with amastigotes obtained from the spleen of infected animals. Briefly, hamsters infected with *L*. *infantum chagasi* were euthanized no later than 60–70 days post-infection. The spleens were removed and triturated in PBS using a glass tissue homogenizer. Spleen homogenates were used to infect young male hamsters and this procedure was performed once a month to continuously maintain the strain in animals. Spleen smears were prepared on microscopic slides and parasite burden was quantified by optical microscopy as described in the section “Quantification of spleen parasite burden by optical microscopy”.

### Generation of the *L*. *infantum chagasi* line expressing luciferase (Lc-LUC)

The modified *Photinus pyralis* luciferase open reading frame (ORF) from plasmid *pLUC2* [15] was amplified with primers Luc2For (5’-GCGGGATCCATGGAAGATGCCAAAAACATTAAG-3’) and Luc2Rev (5’-CACGCGCATACATTCACGGCGTTACACGGCGATCTTGCCGC-3’). A fragment of the *Leishmania enrietti* α-tubulin 3’ untranslated region (3’ UTR) was amplified from plasmid *pSPαHYGα* [16] with primers αTubForLuc2 (5’-GCGGCAAGATCGCCGTGTAACGCCGTGAATGTATGCGCGTG-3’) and αTubRev*Bam*HI (5’-GCGGGATCCGGGGAGAGGGATGAGGGGT-3’). To allow for constitutive expression of luciferase in *Leishmania*, the 3’ α-tubulin UTR fragment was linked downstream to the luciferase ORF by overlap PCR and cloned into the *Bam* HI restriction site of the vector *pSSUint* [17]. This vector contains the sequence encoding the hygromycin phosphotransferase gene as the resistance marker and fragments of the *Leishmania* small subunit (SSU) ribosomal DNA (rDNA) at the cassette extremities in order to promote homologous recombination. The resulting plasmid (*pSSUint-Luc2)* was sequenced to confirm the integrity of the insert. The linear cassette containing the sequences of interest was purified from agarose gels after digesting *pSSUint-Luc2* with *Pac* I and *Pme* I. *L*. *infantum chagasi* promastigotes were transfected with 5 μg of the linear digested DNA construct as described [18]. Briefly, 4×10^7^ promastigotes from log-phase cultures were transfected by electroporation at 2,250 V/cm and 500 μF. Cells were then transferred to 10 mL of M199 medium and incubated for 24 h at 25°C. After 24 hours, 32 μg/mL hygromycin was added for selection of mutants. After four passages in liquid medium with hygromycin, mutants were plated on semi-solid M199 medium supplemented with 1.2 μg/mL biopterin, 1% agar, 2% urine and 32 μg/mL hygromycin for clone selection [18]. Integration into the SSU rDNA was confirmed through PCR amplification with primers complementary to sequences inside and outside the transfected cassette. Primers S1 (5’-GATCTGGTTGATTCTGCCAG-3’) and S4 (5’-GATCCAGCTGCAGGTTCACC-3’) [19] anneal to the SSU rDNA sequence flanking the insertion sites and primers Luc2For, Luc2Rev and Luc465–484 (5’-GACCGACTACCAGGGCTTCC-3’) are complementary to the cassette SSU:Luc2αTub. The vectors *pSSU-int* and *pSPαHYGα* were kindly provided by Dr. Tony Aebischer (Robert Koch Institute, Berlin, Germany) and Dr. Marc Oullette (Universite Laval, Quebec, Canada).

### Luciferase *in vitro* assay

Promastigotes of Lc-LUC were harvested at the late log-phase of growth, washed twice and resuspended in phosphate-buffered saline (PBS) (pH 7.2). Parasites were serially diluted and the luciferase assay was performed with One Glo Luciferase Assay System (Promega Corporation) according to the manufacturer’s instructions. Briefly, one volume of reagent was added to 5 volumes of parasite suspension and luminescence was registered in a microplate reader (POLARstar Omega, BMG Labtech, Ortenberg, Germany). Each point was tested in triplicate in at least two independent experiments.

### Determination of half maximal effective concentration (EC_50_) in intracellular amastigotes

Resident macrophages were collected from the peritoneal cavity of BALB/c mice by washing with RPMI-1640 medium (Gibco, Invitrogen Corporation) supplemented with 10% FCS and added to 24-wells plates in round glass cover slips at 4×10^5^/well. Plates were incubated in 5% CO_2_ for 24 h at 37°C. *L*. *Infantum chagasi* amastigotes extracted from the spleen of infected golden hamsters were added to macrophages at a ratio of 10:1 (amastigotes:macrophage). After 24 h, extracellular parasites were removed by washing. Increasing concentrations of pentavalent antimony (N-Methylglucamine antimonate—Glucantime, Sanofi-Aventis, Brazil) and miltefosine (Sigma-Aldrich) were added to infected macrophages and treatment was performed for 120 or 72 hours, respectively. Stock solutions of miltefosine (20 mM) were prepared in sterile distilled water. Glucantime was kindly donated by the Brazilian Ministry of Health. Both drugs were freshly diluted to the final concentration in RPMI-1640 medium immediately before the experiment. Infected macrophages incubated without drugs were used as controls. After the end of drug treatment, cells were fixed in methanol and stained with the Romanovsky type Instant Prov kit (Newprov, Pinhais, PR, Brazil). The percentage of infected cells was determined by counting 200 macrophages in each of the replicates. EC_50_ values were determined from sigmoidal regression of the concentration-response curves using GraphPad Prism 5 software. Each point was tested in duplicate and experiments were performed three times.

### Experimental treatment of Lc-LUC infected hamsters with Sb^V^ and miltefosine

Male hamsters (3 to 5 weeks-old) were obtained from the Instituto de Medicina Tropical de São Paulo of the University of São Paulo, kept in cages with absorbent material and received unlimited food and water.

To establish the infection in hamsters with transgenic parasites, animals were infected with 1.5×10^9^ promastigotes from stationary phase cultures via the intraperitoneal route. In the subsequent experiments, animals were intraperitoneally infected with 10^7^ Lc-LUC amastigotes obtained from the spleen of infected hamsters. Thirty-five days post-infection, parasite burden was quantified in live animals through luciferase detection. Animals were distributed in experimental groups according to the parasite load. At day 40 post-infection, treatment was initiated. Animals received Sb^V^ or miltefosine for 15 or 10 consecutive days, respectively. Parasite burden was quantified at day 56 through luciferase detection and/or microscopic counting of spleen smears.

In the first experiment, Lc-LUC infected hamsters were assigned to groups (n = 6) that were either left untreated or received 50 mg/kg/day Sb^V^ (which corresponds to 85.18 mg/kg/day of Glucantime) intraperitoneally in 200 μL final volume of PBS. The second experiment was performed with groups of Lc-LUC infected hamsters (n = 5) that were left untreated or received 50 mg/kg/day Sb^V^ as described above or 20 mg/kg/day miltefosine by oral gavage in 200 μL final volume. The third experiment was performed with 4 groups (n = 4 or 5) that received 0, 5, 10 or 20 mg/kg/day miltefosine by oral gavage as described above. In this experiment, the animals were followed up for 5 months for survival analysis.

### *In vivo* and *in situ* bioluminescent image quantification

Lc-LUC light emission in live animals was recorded by bioimaging (IVIS Spectrum, Caliper Life Sciences). Previous to the imaging, each animal received 6 mg VivoGlo Luciferin (Promega Corporation) intraperitoneally followed by anesthesia in a 3% isoflurane atmosphere (Cristália). Animals were then transferred to the imaging chamber and kept in a 2.5% isoflurane atmosphere. Total photon emission from a defined region of interest (ROI) was collected using the high resolution (medium binning) mode. The same ROI was applied to all animals. The images were acquired 5 min after luciferin injection. Total photon emission was quantified with Living Image software version 4.3.1 (Caliper Life Sciences) and results were expressed as the number of photons/second/square centimeter/steradian. The photon signal from the abdominal region was presented as a pseudocolor image representing light intensity (red = most intense and blue = least intense) and superimposed on the gray scale reference image [13]. The average background signal was estimated in an uninfected animal and was used to correct the bioluminescent emission by subtraction.

For in situ imaging, Lc-LUC infected hamsters received 6 mg VivoGlo Luciferin (Promega Corporation) intraperitoneally and were anesthetized in a 3% isoflurane atmosphere (Cristália). Animals were euthanized through cervical dislocation, the peritoneal cavity was accessed through the linea alba and the viscera were exposed. Animals were then transferred to the imaging chamber and emitted photons were recorded immediately. Bioluminescent organs were collected and placed in a 24-well plate containing luciferin in PBS and images were acquired immediately.

### Quantification of spleen parasite burden by optical microscopy

Spleen smears were prepared on microscopic slides, stained with the Instant Prov kit (Newprov, Pinhais, PR, Brazil) and examined under optical microscopy to identify *Leishmania* amastigote forms. Results were expressed as Leishman Donovan Units (LDU) corresponding to the number of amastigotes per 1000 nucleated cells multiplied by the organ weight in grams.

### Statistical analysis

Data on parasite burden was analyzed for statistical significance by One Way ANOVA, followed by the Tukey post-test. Statistical analyses were performed using GraphPad Prism 5 software. A result was considered significant at *p*<0.05.

## Results

### Generation of Lc-LUC

**Fig 1:**
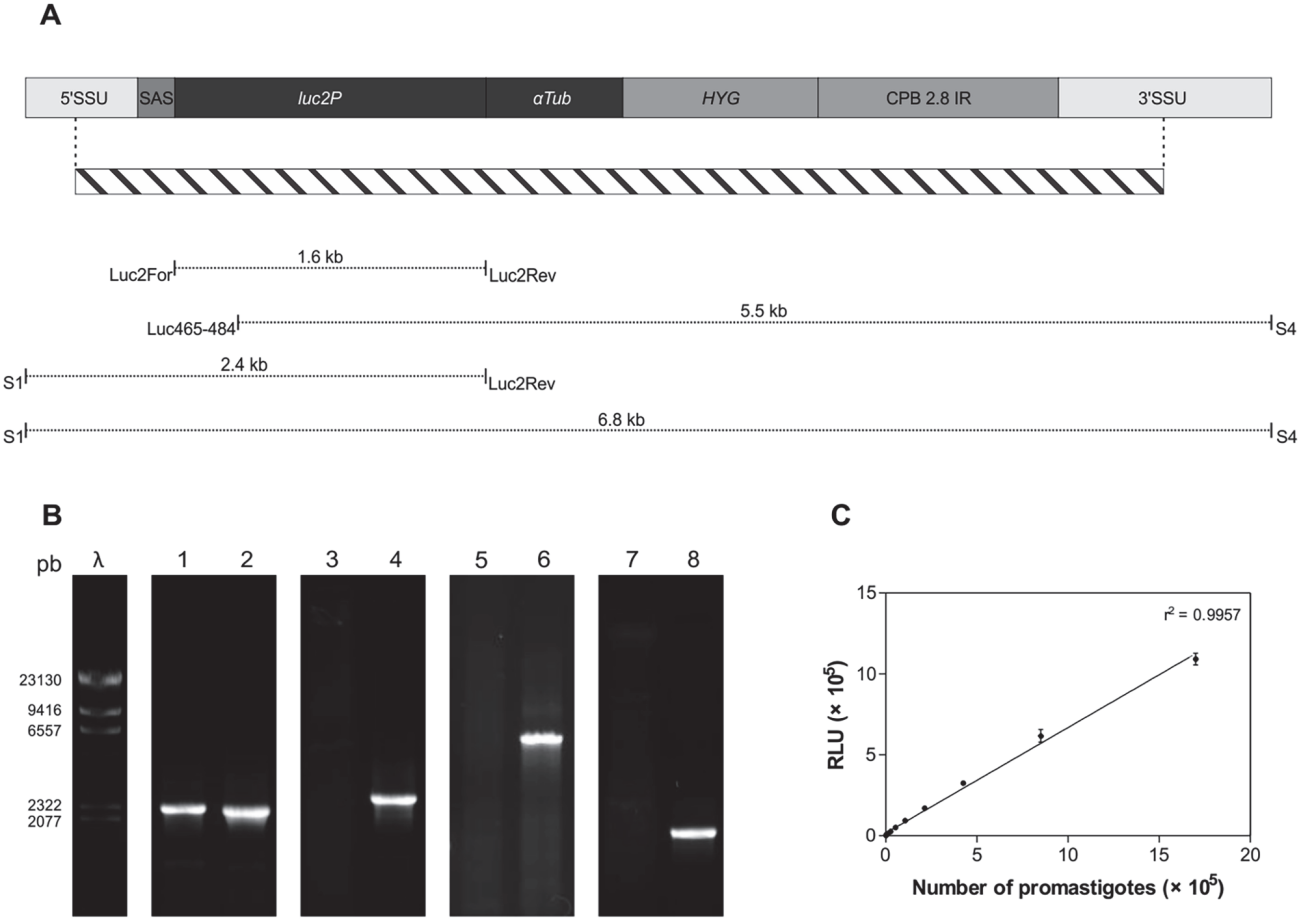
Generation of the *L*. *infantum chagasi* line expressing luciferase (Lc-LUC). (**A**) Schematic representation of the SSU rDNA locus after integration of the linearized construct derived from *pSSUint-Luc2*. SSU: small subunit; SAS: splice acceptor site; LUC2: *luc2P* coding sequence; *αTub* IR: intergenic region of the *L*. *enrietii α-tubulin* gene (El Fadili et al., 2002); HYG: hygromycin phosphotransferase gene; *CPB-2*.*8* IR: *L*. *mexicana CPB 2*.*8* intergenic region. The hatched bar indicates the transfected cassette. Position of primers and size of amplified fragments are indicated by dotted lines. (**B**) Size separation of PCR products obtained from Lc-WT (lanes 1, 3, 5 and 7) and Lc-LUC (lanes 2, 4, 6 and 8) genomic DNA with primers S1 and S4 (1 and 2), S1 and Luc2Rev (3 and 4), Luc465–484 and S4 (5 and 6), Luc2For and Luc2Rev (7 and 8). (**C**) Correlation between luciferase activity and number of Lc-LUC promastigotes. Parasites were serially diluted and luminescence was measured using a microplate reader. Results are the mean and standard deviation of triplicate determinations from a representative experiment. RLU: relative luminescence units.

*L*. *infantum chagasi* line expressing luciferase (Lc-LUC) was obtained by transfection of a cassette containing the *luc2P* gene flanked by ribosomal DNA sequences which directed homologous recombination into the *L*. *infantum chagasi* ribosomal DNA locus (Fig 1A). After clone selection, integration was confirmed by PCR amplification of the flanking regions using primers complementary to sequences outside (primers S1 and S4) and inside the transfected cassette (primers Luc2For and Luc2Rev) (Fig 1A-B). Amplification reactions performed on the transgenic line with primers S1/Luc2Rev and Luc465–484/S4 generated 2.4 kb and 5.5 kb fragments, respectively. The positive amplification of the *luc2P* gene was demonstrated in the transgenic line by the detection of a 1.6 kb amplified fragment. Amplifications with these sets of primers occurred only in the transgenic line and showed the expected size, confirming integration into the SSU rDNA (Fig 1B). Primers S1 and S4 [19] amplified the SSU rDNA in both the wild-type and transgenic line resulting in a 2.2 kb fragment. The amplification of a fragment of 6.8 kb in size resulting from the rDNA cistron where the integration occurred was not observed, most likely because of the relative abundance of the normal cistron, highly repeated in the genome.

Luciferase expression in the transgenic lines was confirmed by light production upon addition of luciferin. A linear correlation between the number of promastigotes and emitted light was found, as shown in Fig 1C, indicating that luciferase activity can be used to assess parasite numbers with confidence.

Transgenic (Lc-LUC) and wild-type (Lc-WT) promastigotes exhibited indistinguishable growth curves, achieving the stationary phase on the fourth day of culture (S1 Fig). In vitro susceptibility of the transgenic line to Sb^V^ and miltefosine was evaluated. The calculated EC_50_ values for Lc-LUC intracellular amastigotes were 111.0 μg/mL (confidence interval 95% = 94.6 to 130.3) and 4.4 μM (confidence interval 95% = 3.3 to 5.9), for Sb^V^ and miltefosine, respectively. The EC_50_ values for Lc-WT intracellular amastigotes were 110.0 μg/mL (confidence interval 95% = 76.68 to 157.9) and 3.88 μM (confidence interval 95% = 3.02 to 4.99), for Sb^V^ and miltefosine, respectively. These values are in accordance with previously published data on *L*. *infantum chagasi* susceptibility to these drugs [7].

### Detection of luciferase in hamsters infected with Lc-LUC

The first experimental infection in hamsters was obtained by intraperitoneal injections of Lc-LUC stationary phase promastigotes. Clinical signs of disease were observed 2–4 months post-infection, when amastigotes were recovered from spleen homogenates. This first set of infected animals served as a source of amastigotes which were pooled and used to infect hamsters for the next experiment. Parasite load in the spleen was determined by LDU and the following infections were achieved by intraperitoneal inoculation of 10^7^ amastigotes per animal.

After establishing the infection in vivo, we performed a pilot experiment to determine the best conditions for hamster imaging. Different doses of luciferin and various capture times were tested. We found that luminescence could be efficiently visualized after 5–10 minutes of administering 6 mg luciferin per animal. One month after infection, animals showed widespread light emission in the abdominal and/or pelvic region disclosing different patterns of parasite dissemination (S2 Fig). Bioluminescence in situ, in an euthanized animal with the peritoneal region exposed is also shown (S2 Fig). Subsequent dissection allowed observation of bioluminescent parasites in different tissues and organs, such as the epididymis and adipose tissue (S3 Fig). Based on this heterogeneous pattern, we defined that the region of interest (ROI) to be used in parasite burden determinations should comprise the whole abdominal and pelvic regions.

### Luciferase as a tool to evaluate treatment efficacy

**Fig 2:**
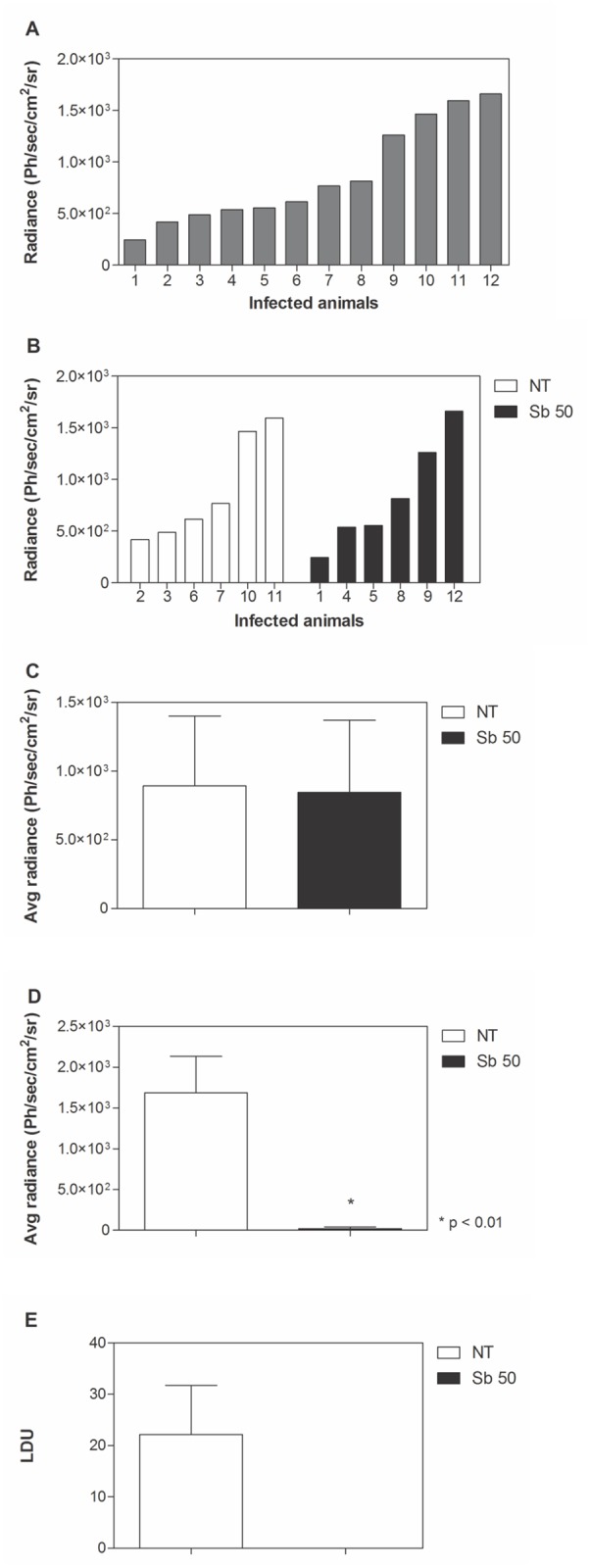
Treatment of Lc-LUC infected hamsters with Sb^V^. Hamsters were infected with 10^7^ Lc-LUC amastigotes. Thirty-five days post-infection, bioimaging was performed in individual hamsters to quantify parasite burden (**A**). Animals were then distributed into experimental groups with equivalent mean parasite burdens (**B**, **C**). At day 40, treatment was initiated with 50 mg/kg/day Sb^V^ (Sb 50) for 15 consecutive days. Parasite burden was quantified at day 56 through luminescence detection (**D**) and by microscopic counting of spleen smears, expressed as Leishman Donovan Units (LDU) (**E**). Ph/sec/cm^2^/sr: photons per second per square centimeter per steradian. Asterisks indicates significant differences in comparison with the untreated group (NT).

Treatment of infected animals with Sb^V^ was used as a proof-of-concept study to validate this model as a tool to evaluate drug activity in hamsters with VL. Before treatment, parasite load was quantified by bioimaging (Fig 2A). A large dispersion of parasite burden was noted between the animals. Emitted light measurements allowed the allocation of animals into experimental groups (Fig 2B) with similar mean radiance (Fig 2C). Treatment was initiated 40 days post-infection and animals received 50 mg/kg/day of Sb^V^ for 15 consecutive days. At the end of treatment (56 days post-infection), parasite burden was quantified by bioimaging (Fig 2D). Thereafter, animals were euthanized and each spleen was used to imprint the organ in glass slides for LDU determination (Fig 2E). Sb^V^ treatment resulted in 98% suppression of bioluminescence when compared with untreated animals (Fig 2D). No parasites were detected by microscopic examination in treated animals (Fig 2E).

### Is miltefosine active against *L*. *infantum chagasi in vivo*?

**Fig 3:**
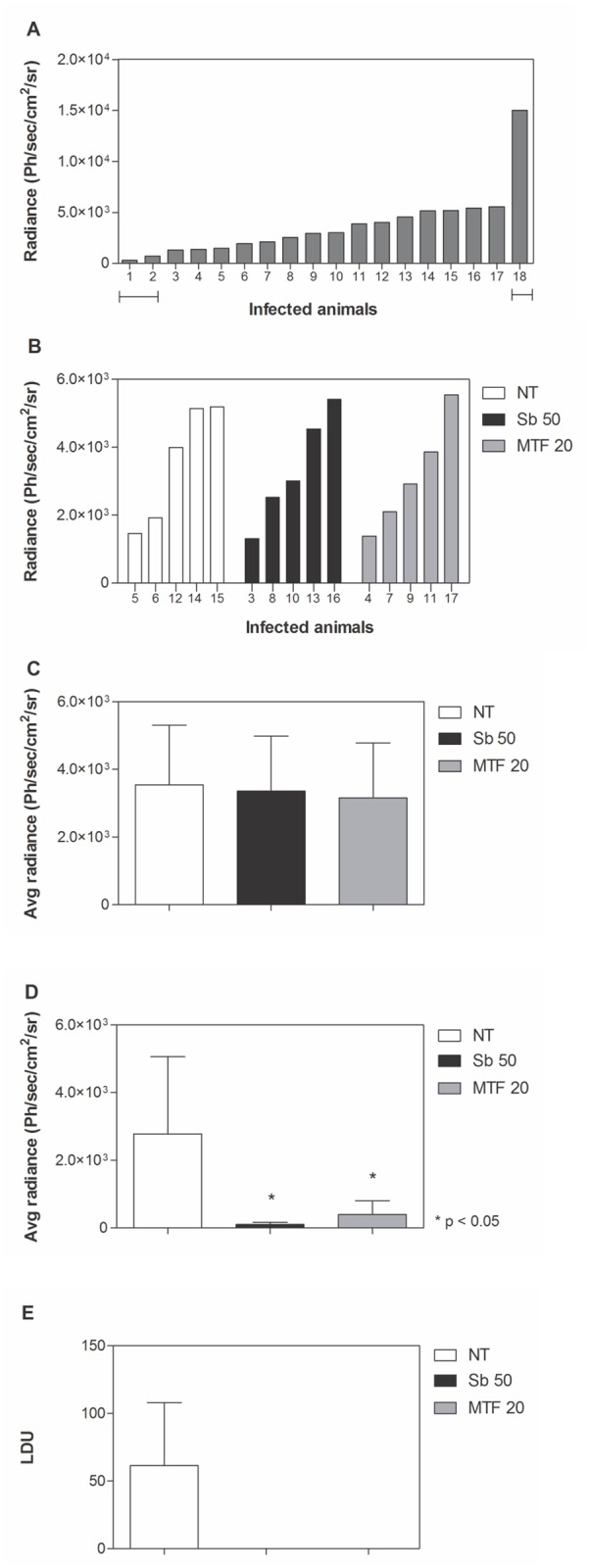
Treatment of Lc-LUC infected hamsters with miltefosine. Animals were infected with 10^7^ Lc-LUC amastigotes and, 35 days post-infection, parasite burden was quantified in live animals through luciferase detection (**A**). Animals with parasite load above or below 4 times the overall average were considered outliers and were excluded from the study (horizontal bars). Animals were divided into equivalent experimental groups according to the parasite load (**B**, **C**). At day 40, treatment started with 50 mg/kg/day Sb^V^ (Sb 50) or 20 mg/kg/day miltefosine (MTF 20) for 15 or 10 consecutive days, respectively. Parasite burden was quantified at day 56 through luciferase detection (**D**) or microscopic counting of spleen smears (**E**), expressed as Leishman Donovan Units (LDU). Ph/sec/cm^2^/sr: photons per second per square centimeter per steradian. Asterisks indicates significant differences in comparison with the untreated group (NT).

This new experimental model was then used to evaluate the efficacy of miltefosine in Lc-LUC infected hamsters. In this experiment, treatment with miltefosine was compared with Sb^V^. Similarly to the first experiment, parasite load was quantified by bioimaging before treatment initiation (Fig 3A). Animals with parasite loads above or below 4 times the overall average of the whole group were considered outliers and excluded from the experiment. Parasite burden based upon light emission was used to distribute animals evenly between three treatment groups (Fig 3B) which therefore had comparable mean levels of infection (Fig 3C).

Treatment was initiated 40 days post-infection and animals received 50 mg/kg/day of Sb^V^ for 15 consecutive days or 20 mg/kg/day of miltefosine for 10 consecutive days. At the end of treatment (56 days post-infection), parasite load was quantified by bioimaging (Fig 3D) and LDU determination (Fig 3E).

According to the bioluminescence quantification (Fig 3D), Sb^V^ resulted in 96% suppression of parasite burden when compared with untreated animals, while treatment with miltefosine resulted in 86% suppression. Parasite load determined through LDU indicated absence of detectable parasites in animals treated with both miltefosine and Sb^V^ (Fig 3E). There were no significant differences in parasite burden between Sb^V^ and miltefosine-treated groups quantified by bioimaging.

### Estimation of miltefosine ED_50_ in Lc-LUC infected hamsters and follow-up of animal survival

**Fig 4:**
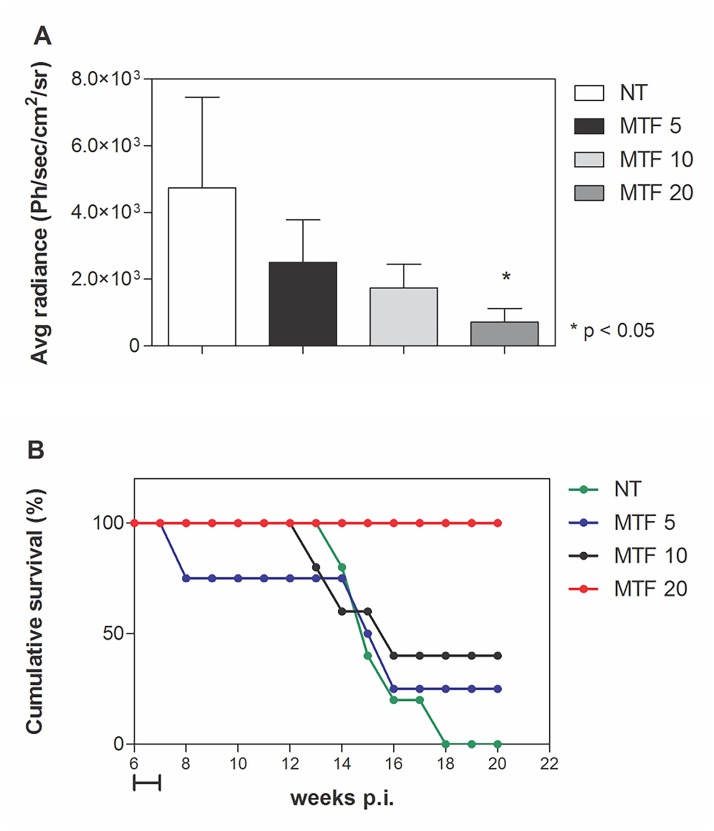
Evaluation of miltefosine’s dose-response in Lc-LUC infected hamsters and follow-up of animal survival. Hamsters were infected with Lc-LUC amastigotes and, starting 40 days post-infection, animals received 5 mg/kg/day (MTF 5), 10 mg/kg/day (MTF 10) or 20 mg/kg/day (MTF 20) miltefosine for 10 consecutive days. Parasite burden was quantified 56 days after infection through luciferase detection (**A**). Asterisks indicate *p* < 0.05 in comparison with the untreated group (NT). Follow-up of animal survival in untreated and miltefosine-treated groups (**B**). The horizontal bar indicates the period of miltefosine administration. Ph/sec/cm^2^/sr: photons per second per square centimeter per steradian.

Having determined that miltefosine was as effective as Sb^V^ in the early stage after treatment, we were interested in determining the effective dose and long term effects of miltefosine in *L*. *infantum chagasi* infections. As in the previous experiments, groups were based on parasite burden determination before treatment (S4 Fig). Miltefosine doses used were 5, 10 and 20 mg/kg/day, which resulted in 47, 63 and 85% reduction in parasite burden compared to the control untreated group, as determined by bioluminescence (Fig 4A). Plotting normalized values on a non-linear regression curve resulted in an ED_50_ of 6.1 mg/kg/day. Bioluminescence imaging of animals from untreated and miltefosine-treated groups at the end of treatment is shown in S5 Fig

To assess the long term drug-efficacy, animals were followed-up for 20 weeks (18 weeks after treatment interruption). The infection led to death in 100% of untreated animals 18 weeks post-infection. Miltefosine-treated animals survived longer as compared with untreated animals in a dose-dependent manner. In the group treated with the highest total dose of miltefosine (20 mg/kg/day), we observed 100% animal survival 20 weeks post-infection (Fig 4B).

## Discussion

We report here the generation of recombinant luciferase-expressing *L*. *infantum chagasi* parasites and their use to quantify parasite load in vivo in infected hamsters. The hamster model is used to study VL because it reproduces the clinical course and pathology of the disease, as seen in humans and dogs [10]. Results presented here contribute to the study of new alternatives for VL treatment through the use of a chronic infection model.

Studies to determine drug efficacy in experimental leishmaniasis involve multiple technical difficulties such as the need for a large number of animals which should be maintained for long periods. Quantification of parasite load is commonly done by limiting dilution protocols or by microscopic examination of slides prepared by imprinting of the infected organs. These techniques involve enormous variability, are laborious and time demanding. Furthermore, spread of parasites to an unexpected site of infection may be missed because the infected tissue is not harvested or analyzed. A more efficient method for quantifying parasite load in vivo would help to overcome these drawbacks.

Recently, the use of firefly luciferase to detect transgenic *Leishmania* has provided many advantages over conventional methods. Bioluminescence allows the detection of live parasites and can be performed repeatedly. Quantification of bioluminescence is not only sensitive but also more rapid than culture-based techniques and can be used to monitor the efficacy of antileishmanial drugs in animal models [10, 13].

Various recombinant parasites carrying a reporter gene as an episomal copy are currently available, as reviewed by [10]. However, for prolonged growth in the absence of drug selection, such as within animal models, quantitation of parasites is more reliable when the gene encoding luciferase is stably integrated into the parasite genome. In fact, when reporters are part of plasmids, the relative output of the reporter may depend on the copy number of the transfected plasmid, which varies from cell to cell, resulting in different levels of expression [20]. In order to circumvent this problem, in the present work, the *luc2P* gene was integrated in the *L*. *infantum chagasi* rDNA locus.

As mentioned previously, luminescent parasites can be quantified repeatedly in the same animal. Genetic variability in hamsters is the cause of considerable discrepancy in VL progression, as is the case in humans and dogs. Individual parasite burdens in individual infected animals were used to distribute them between different treatment groups so that the mean parasite burdens were equivalent before treatment initiation. This procedure brings substantial progress in experimentation with hamsters, allowing drug efficacy to be evaluated with greater reliability.

We also observed that infected animals showed a heterogeneous pattern of parasite dissemination. This demonstrates the inherent variability of experimentation with hamsters and the possibility of parasite spread to different sites. Based on this, we defined a ROI corresponding to the whole abdominal and pelvic region, including in the analysis an extensive area where parasite could be found. Peritoneum bioluminescence, distinct from spleen bioluminescence, was reported by [14] in luciferase-expressing *L*. *infantum* infected BALB/c. The presence of *L*. *infantum* in the epididymis of infected hamsters and dogs was also previously reported [21–23]. However, these unusual sites of infection are commonly ignored in drug efficacy assays.

It has to be said that the parasite distribution observed herein may be the result of the intraperitoneal infection route used in this study. The choice of this infection route was justified by the difficulty of intravenous access in hamsters, which lack usable tails. While natural infection upon sand fly feeding results in parasite inoculation into dermal tissue and capillary blood the intraperitoneal route may artificially create extensive abdominal dissemination. It remains to be investigated whether intracardiac inoculation would result in less disseminated parasite colonization.

We found that transgenic and parental lines presented similar growth rates (S1 Fig) and in vitro susceptibility to Sb^V^ and miltefosine, with EC_50_ values in the same range as previously published data [7]. Transfected parasites were not attenuated in vivo compared with the parental strain, leading to animal death 3–5 months post-infection (Fig 4). We found that Sb^V^ was able to reduce parasite load in Lc-LUC infected hamsters to very low levels, as reported previously in infections with the wild type parasites of the same strain [24, 25]. So, in all aspects evaluated, the luciferase expressing line behaved similarly to the parental parasites.

In order to validate the use of bioluminescence as a quantitative tool for parasite load determination, we performed the experimental treatment of Lc-LUC-infected hamsters with antileishmanial drugs and bioluminescence results were compared with the classical method of LDU determination. Interestingly, luciferase detection was more sensitive than LDU: while LDU indicated parasite clearance in hamsters treated with antimony and miltefosine, bioimaging revealed the persistence of parasites in small numbers. We have previously shown that bioluminescence data were in accordance with limiting dilution and clinical parameters evaluated after experimental treatment with amphotericin B when using luminescent *L*. *amazonensis* in infected mice [13]. Our present work confirms that bioluminescence is a reliable tool for parasite load determination.

Miltefosine was as effective as Sb^V^ in the treatment of Lc-LUC infections in hamsters. Data on the efficacy of miltefosine in *L*. *infantum*-infected hamsters are limited. In one study [9], the authors used an early curative model where treatment was initiated 21 days post-infection and parasite burden determined 35 days post-infection. In these settings, milfefosine treatment resulted in 61 and 99% reduction in parasite burden in the spleen when animals were treated with total doses of 100 and 200 mg/kg, respectively. Here, we used a late curative therapeutic approach, as we started treatment 40 days post-infection and parasite burden was estimated 56 days post-infection. We chose the doses of 10 and 20 mg/kg/day (total dose of 100 and 200 mg/kg), as previously reported [9], and included a lower dose of 5 mg/kg/day, in order to estimate miltefosine’s ED_50_. In our model, doses of 5, 10 and 20 mg/kg/day miltefosine resulted in 47, 63 and 85% reduction in parasite burden. These differences in parasite reduction could be explained by the fact that we assessed parasite load in an extensive area, corresponding to the abdominal and pelvic region, while the data from literature refers to parasite load in specific organs, determined by LDU [9]. Furthermore, differences were expected due to the dissimilar protocols used in both experiments (early versus late curative model).

We showed that miltefosine is effective in the experimental treatment of *L*. *infantum chagasi*-infected hamsters, as demonstrated by the reduction in parasite burden in a dose-dependent manner and by prolongation of animal survival. However, we found that, even for the highest dose used, clinical response did not reflect sterile cure. The same is true for antimony-treated hamsters and it may still reflect clinical cure. On the other hand, it is possible that the remaining parasites may lead to disease recurrence. In a study performed with dogs naturally infected with *L*. *infantum chagasi*, miltefosine treatment resulted in improvement of clinical symptoms but did not result in parasitological clearance [26]. Additional studies are needed to ascertain if total doses higher than 200 mg/kg could result in parasite clearance in the hamster model. In any case, results shown here indicated that *L*. *infantum chagasi* infections are responsive to miltefosine treatment and, at least in the hamster model, resulted in 100% survival in treated animals until 20 weeks post-infection, as opposed to 100% mortality in the control group.

Data presented here also indicates that the use of luminescent *L*. *infantum chagasi* is a reliable alternative for parasite burden quantification in hamsters. This tool has several advantages such as the possibility of assessing the progress of infection in the same animal and the benefit of estimating parasite load before and after drug treatment. The possibility of distributing animals in equivalent groups is an important advantage, especially when working with heterogenic animals, as is the case of hamsters. This model may be useful for the study of pathogenesis and healing processes in hamsters, allowing the dissection of parasite persistence.

## Supporting Information

S1 Fig Growth curves of Lc-WT and Lc-LUC promastigotes. Promastigotes were grown in 25 cm^2^ tissue culture flasks containing M199 medium supplemented with 10% heat-inactivated fetal calf serum, 0.25% hemin and 2% sterile male human urine at 25°C. Aliquots were counted using an haemocytometer every 24 hours. Standard deviation of the mean of triplicate cultures is shown.
(TIF)

S2 Fig Localization of bioluminescent parasites in Lc-LUC infected hamsters. Hamsters were infected via the intraperitoneal route with 10^7^ Lc-LUC amastigotes obtained from the spleen of infected hamsters and bioluminescence was measured one month post-infection. In vivo images of distinct Lc-LUC infected hamsters (**A-K**) and in situ (L) quantification of luminescent parasites. AR: average radiance, given in Ph/sec/cm^2^/sr (photons per second per square centimeter per steradian).
(TIF)

S3 Fig Bioluminescent Lc-LUC in the epididymis and adipose tissue. Bioluminescent organs of Lc-LUC-infected hamsters were collected and placed in a 24-well plate containing luciferin in PBS and images were acquired immediately. Epididymis and adipose tissue from three distinct infected hamsters are shown. Ph/sec/cm^2^/sr: photons per second per square centimeter per steradian.
(TIF)

S4 Fig Parasite load quantification through bioluminescence before miltefosine treatment. Animals were infected with 10^7^ Lc-LUC amastigotes and, at day 35, parasite burden was quantified in live animals through luciferase detection (**A**). Animals with parasite load above or below 4 times the overall average were considered outliers and were excluded from the study (horizontal bars). Animals were divided into four equivalent experimental groups according to the parasite load, corresponding to untreated group (NT) or miltefosine-treated groups (**B**, **C**). Starting 40 days post-infection, animals received 5 mg/kg/day (MTF 5), 10 mg/kg/day (MTF 10) or 20 mg/kg/day (MTF 20) miltefosine for 10 consecutive days in order to estimate miltefosine ED_50_, as shown in Fig 4. Ph/sec/cm^2^/sr: photons per second per square centimeter per steradian.
(TIF)

S5 Fig Bioluminescence imaging after miltefosine treatment. Animals were infected with 10^7^ Lc-LUC amastigotes and, 40 days post-infection, animals received 5 mg/kg/day (MTF 5), 10 mg/kg/day (MTF 10) or 20 mg/kg/day (MTF 20) miltefosine for 10 consecutive days. 56 days after infection, parasite burden was quantified in live animals through luciferase detection. Untreated and uninfected animals were used as positive and negative controls, respectively. The region of interest (ROI) is circled in red. AR: average radiance, given in Ph/sec/cm^2^/sr (photons per second per square centimeter per steradian).
(TIF)
