# Supplementary figures and images for: Generation of Luciferase-Expressing Leishmania infantum chagasi and Assessment of Miltefosine Efficacy in Infected Hamsters through Bioimaging
Source: PLoS Negl Trop Dis. 2015 Feb 13;9(2):e0003556. doi: 10.1371/journal.pntd.0003556 (PMC4332486; doi:10.1371/journal.pntd.0003556)

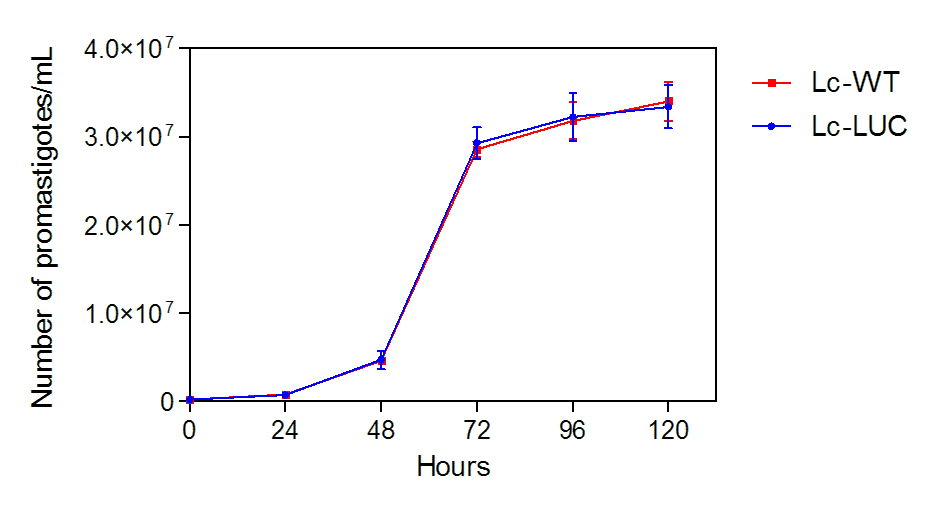

Supplement: S1 Fig — Promastigotes were grown in 25 cm2 tissue culture flasks containing M199 medium supplemented with 10% heat-inactivated fetal calf serum, 0.25% hemin and 2% sterile male human urine at 25°C. Aliquots were counted using an haemocytometer every 24 hours. Standard deviation of the mean of triplicate cultures is shown. (TIF) [file pntd.0003556.s001.tif]

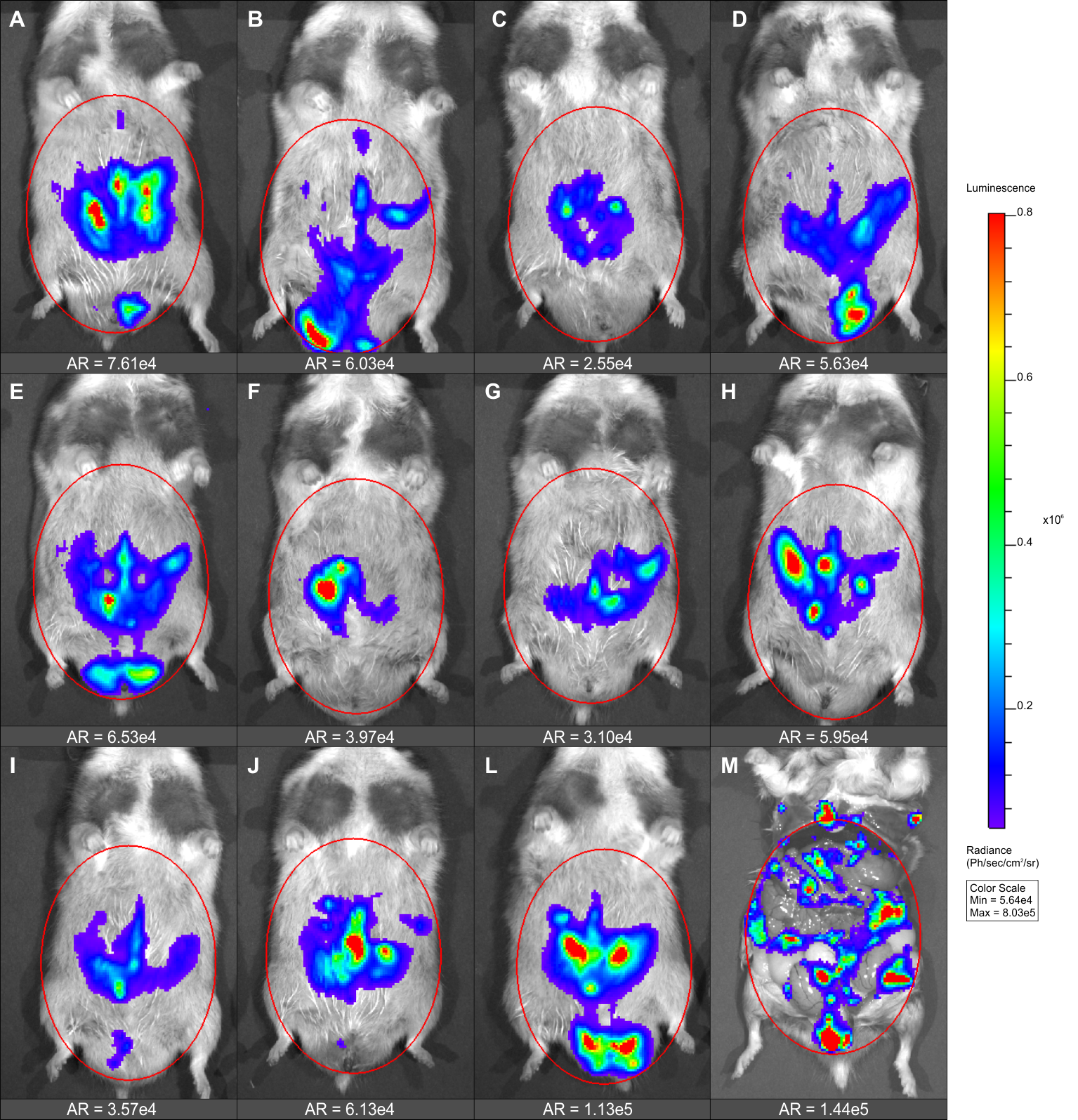

Supplement: S2 Fig — Hamsters were infected via the intraperitoneal route with 107 Lc-LUC amastigotes obtained from the spleen of infected hamsters and bioluminescence was measured one month post-infection. In vivo images of distinct Lc-LUC infected hamsters (A-K) and in situ (L) quantification of luminescent parasites. AR: average radiance, given in Ph/sec/cm2/sr (photons per second per square centimeter per steradian). (TIF) [file pntd.0003556.s002.tif]

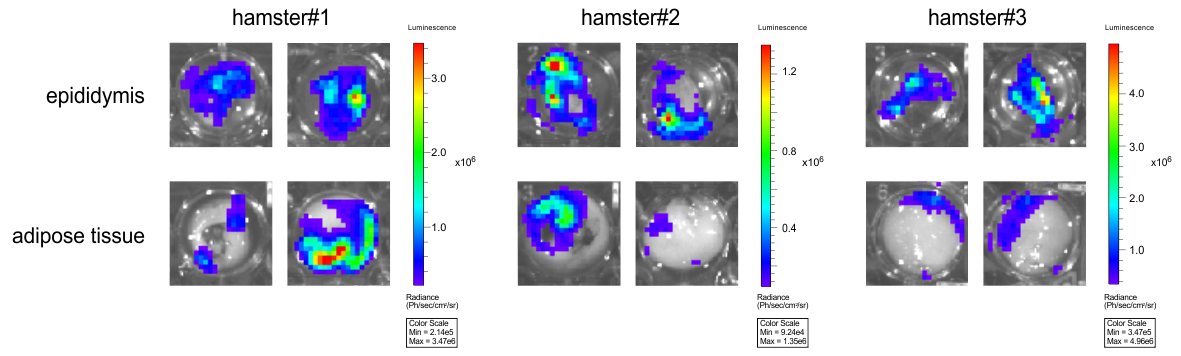

Supplement: S3 Fig — Bioluminescent organs of Lc-LUC-infected hamsters were collected and placed in a 24-well plate containing luciferin in PBS and images were acquired immediately. Epididymis and adipose tissue from three distinct infected hamsters are shown. Ph/sec/cm2/sr: photons per second per square centimeter per steradian. (TIF) [file pntd.0003556.s003.tif]

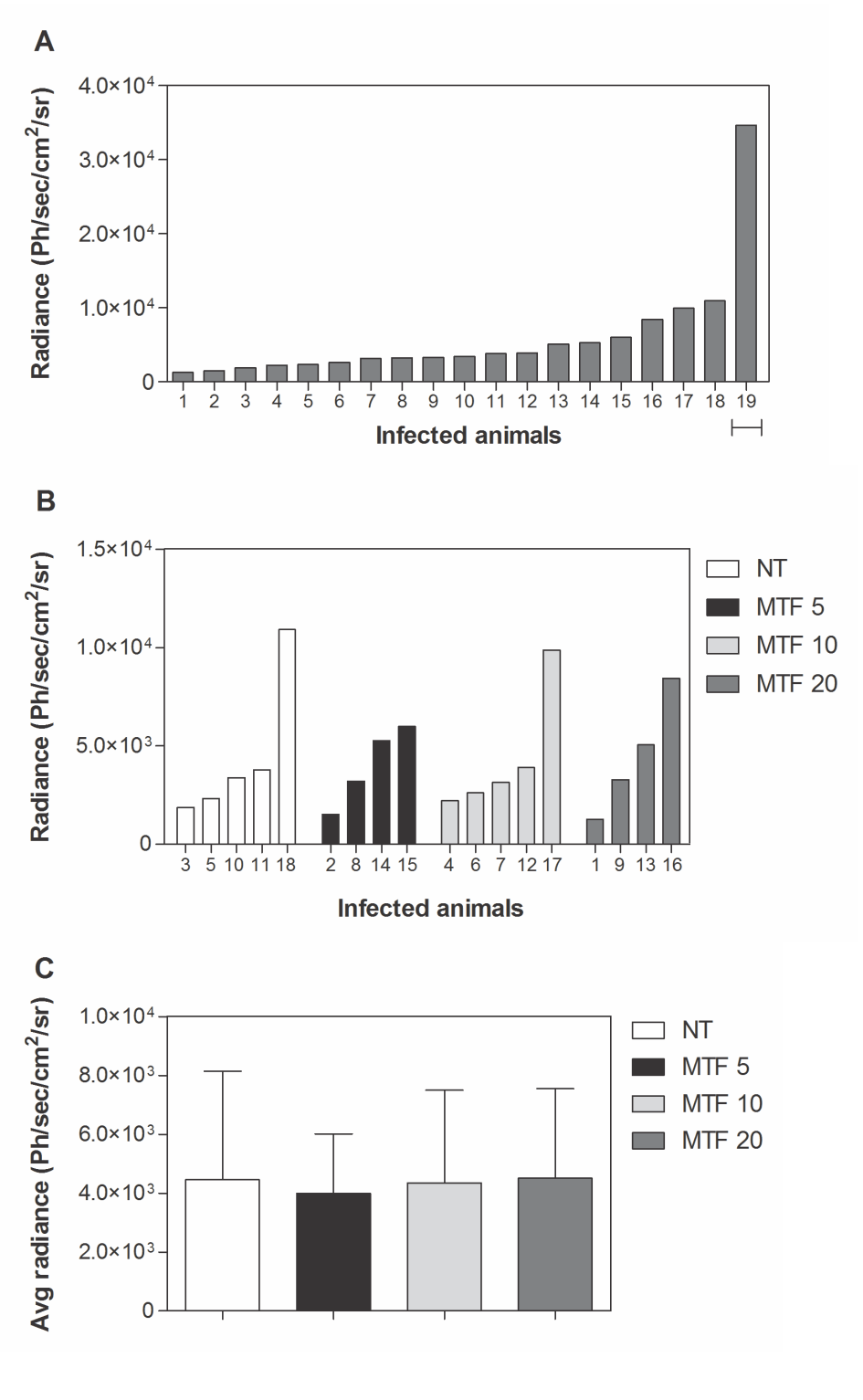

Supplement: S4 Fig — Animals were infected with 107 Lc-LUC amastigotes and, at day 35, parasite burden was quantified in live animals through luciferase detection (A). Animals with parasite load above or below 4 times the overall average were considered outliers and were excluded from the study (horizontal bars). Animals were divided into four equivalent experimental groups according to the parasite load, corresponding to untreated group (NT) or miltefosine-treated groups (B, C). Starting 40 days post-infection, animals received 5 mg/kg/day (MTF 5), 10 mg/kg/day (MTF 10) or 20 mg/kg/day (MTF 20) miltefosine for 10 consecutive days in order to estimate miltefosine ED50, as shown in Fig 4. Ph/sec/cm2/sr: photons per second per square centimeter per steradian. (TIF) [file pntd.0003556.s004.tif]

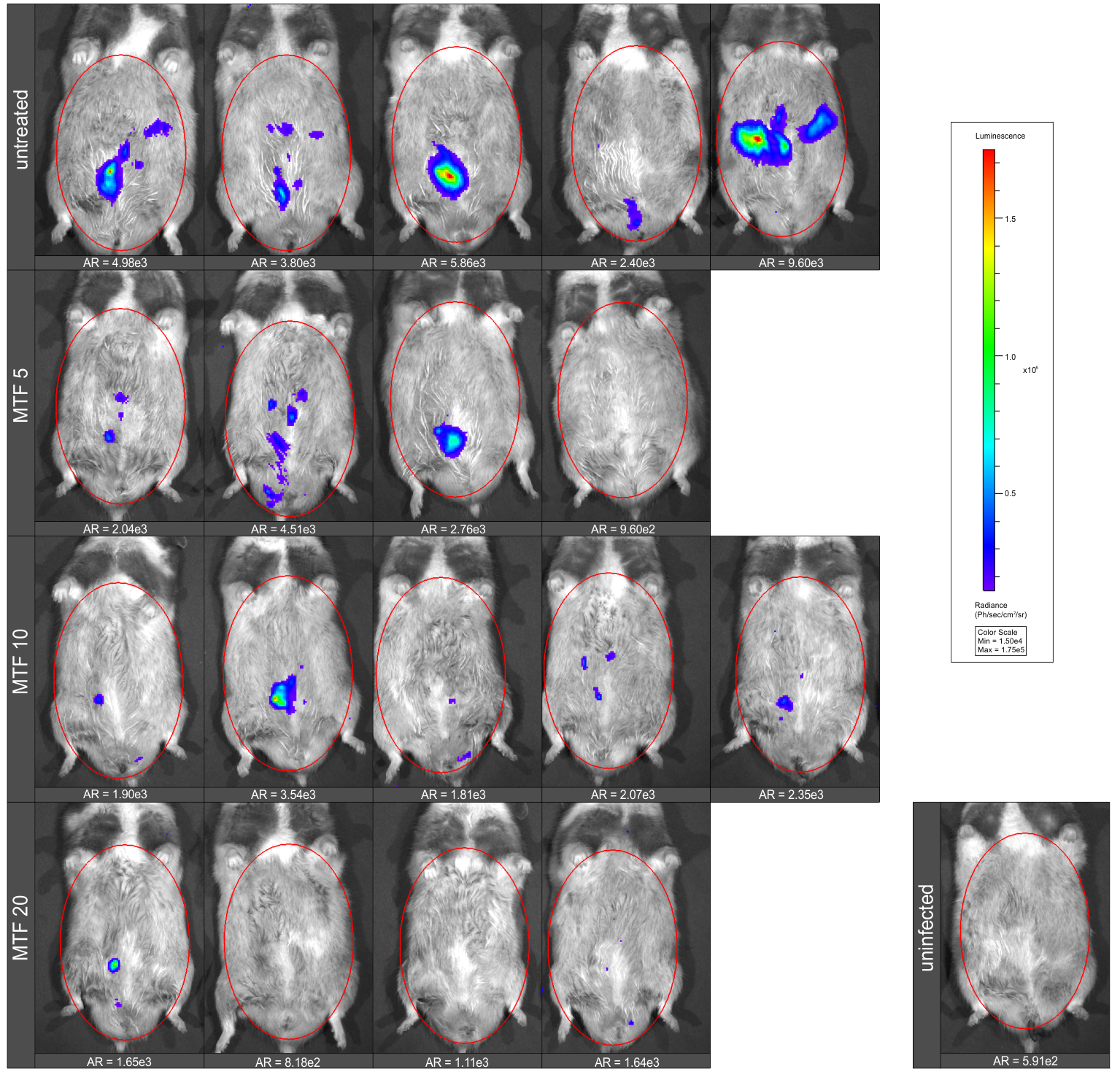

Supplement: S5 Fig — Animals were infected with 107 Lc-LUC amastigotes and, 40 days post-infection, animals received 5 mg/kg/day (MTF 5), 10 mg/kg/day (MTF 10) or 20 mg/kg/day (MTF 20) miltefosine for 10 consecutive days. 56 days after infection, parasite burden was quantified in live animals through luciferase detection. Untreated and uninfected animals were used as positive and negative controls, respectively. The region of interest (ROI) is circled in red. AR: average radiance, given in Ph/sec/cm2/sr (photons per second per square centimeter per steradian). (TIF) [file pntd.0003556.s005.tif]
